# CancellationTools: All-in-one software for administration and analysis of cancellation tasks

**DOI:** 10.3758/s13428-014-0522-7

**Published:** 2014-11-08

**Authors:** Edwin S. Dalmaijer, Stefan Van der Stigchel, Tanja C. W. Nijboer, Tim H. W. Cornelissen, Masud Husain

**Affiliations:** 1 Department of Experimental Psychology, University of Oxford, Tinbergen Building, 9 South Parks Road, Oxford, OX1 3PS UK; 2 Department of Experimental Psychology, Helmholtz Institute, Utrecht University, Utrecht, The Netherlands; 3 Rudolf Magnus Institute of Neuroscience and Center of Excellence for Rehabilitation Medicine, University Medical Center Utrecht and Rehabilitation Center de Hoogstraat, Utrecht, The Netherlands; 4 Nuffield Department of Clinical Neurosciences, University of Oxford, Oxford, UK

**Keywords:** Spatial attention, Visual search, Neglect syndrome, Cancellation tasks, Computerized testing

## Abstract

In a cancellation task, a participant is required to search for and cross out (“cancel”) targets, which are usually embedded among distractor stimuli. The number of cancelled targets and their location can be used to diagnose the neglect syndrome after stroke. In addition, the organization of search provides a potentially useful way to measure executive control over multitarget search. Although many useful cancellation measures have been introduced, most fail to make their way into research studies and clinical practice due to the practical difficulty of acquiring such parameters from traditional pen-and-paper measures. Here we present new, open-source software that is freely available to all. It allows researchers and clinicians to flexibly administer computerized cancellation tasks using stimuli of their choice, and to directly analyze the data in a convenient manner. The automated analysis suite provides output that includes almost all of the currently existing measures, as well as several new ones introduced here. All tasks can be performed using either a computer mouse or a touchscreen as an input device, and an online version of the task runtime is available for tablet devices. A summary of the results is produced in a single A4-sized PDF document, including high quality data visualizations. For research purposes, batch analysis of large datasets is possible. In sum, CancellationTools allows users to employ a flexible, computerized cancellation task, which provides extensive benefits and ease of use.

## Introduction

Almost half of all stroke patients initially suffer from impaired attention (Lesniak et al., 2008). One of the most severe stroke-induced attention deficits is hemispatial neglect, a syndrome where patients disregard what happens towards contralesional space. It occurs in 25–50 % of stroke victims (Appelros et al., 2002; Buxbaum et al., 2004; Nijboer et al., 2013a), predominantly after damage to the right hemisphere (Ringman et al., 2004). Stroke patients suffering from neglect are hospitalized longer and face profound problems in daily life (Nijboer et al., 2013b; Nys et al., 2005). Although spontaneous recovery occurs, about 30–40 % of individuals with neglect still suffer from the syndrome after a year (Nijboer et al., 2013a, b). Importantly, neglect is associated with many negative factors, for example it appears to have a suppressive effect on upper-limb motor recovery (both synergism and strength) especially over the first ten weeks post-stroke (Nijboer et al., 2014).

Because of its severity, it is important that good tools are available to diagnose the neglect syndrome, and to support research on potential rehabilitation methods. One type of test that is widely used for assessment measures multitarget visual search. Such cancellation tasks require participants to cross out (“cancel”) all stimuli of a certain type, often while ignoring stimuli of all other types (distractors). These search tasks have gained immense popularity in cognitive neuropsychology, and have proven their worth both in clinical and research environments.

Cancellation performance is not only a measure of interest in patient groups, but in other sets of participants as well. For example, a recent study on a wide age range of healthy adults described search patterns on cancellation tasks in a qualitative manner (e.g., “*horizontal left*-*to*-*right*”), and concluded that no significant differences exist between different age groups (Warren et al., 2008). However, this investigation lacked more sensitive measures of search organization that have been shown to improve with age in children (Woods et al., 2013). Healthy elderly people tested two years before dementia require significantly more time to complete a cancellation task than elderly individuals who did not develop dementia (Fabrigoule et al., 1998). Differences in performance within demented patients became apparent when tests of a higher attentional load were deployed: patients with Alzheimer's disease performed as accurately as patients with multi-infarct dementia on a low-load cancellation task, but were both less accurate and faster on a cancellation task that required more selective and divided attention (Gainotti et al., 2001). Principal component analysis of a range of neuropsychological tests, including cancellation, indicates there might be a common factor underlying performance deterioration for in the pre-clinical stage of Alzheimer's disease, perhaps associated with a general ability to control cognitive processes (Fabrigoule et al., 1998).

All of the findings summarized above could profitably be extended with more sensitive measures of cancellation performance and search organisation. When diagnosing neglect, the primary measures of cancellation tasks are usually the amount and spatial spread of omissions (non-cancelled targets). However, there is emerging evidence that the neglect syndrome constitutes more than just lateralized deficits (Husain & Rorden, 2003), and deficits of spatial working memory or sustained attention might contribute, for which additional indices of cancellation performance might be helpful.

Numerous measures of general performance, timing, and search strategy that can be derived from cancellation tasks have been suggested in the literature (for an overview, see the section *Supported Measures*). However, data collection for these measures is often performed using labor-intensive and perhaps suboptimal procedures, e.g., frame-by-frame video analysis (Mark et al., 2004; Woods & Mark, 2007), monitoring of “verbal cancellation” (Samuelsson et al., 2002), “observing and recording the predominant search pattern” during a task by a human observer (Warren et al., 2008), or asking patients to change the color of their pencil every 10–15 cancellations (Weintraub & Mesulam, 1988). A more efficient way of analyzing search patterns would be to use a computerized cancellation task, with which cancellation positions and order can be recorded without the risk of human error.

Although the first reports of computerized cancellation software date back 15 years (Donnelly et al., 1999), the currently available packages are very limited in either the number of supported tasks (Donnelly et al., 1999; Wang et al., 2006), or the supported measures (Rorden & Karnath, 2010; Wang et al., 2006), and none of them provide both task presentation and data analysis (CACTS by Wang et al. is reported to be able to do both, but is not available for download). Therefore, most laboratories use custom software and most clinicians still prefer pen-and-paper tests.

Due to the lack of practically useful software, the field is currently in a situation in which ample theoretically valid measures exists (Donnelly et al., 1999; Hills & Geldmacher, 1998; Malhotra et al., 2006; Mark et al., 2004; Rorden & Karnath, 2010; Samuelsson et al., 2002; Warren et al., 2008; Weintraub & Mesulam, 1988), of which most are validated on a small scale in research studies, but very few can be applied on a large scale in clinical practice or research studies due to the aforementioned practical issues.

In the current paper, we present a potential solution: CancellationTools, a package that combines the administration and the analysis of cancellation tasks, supporting almost all types of cancellation tests, and outputting almost all of the currently available research measures. The software is designed to be as user-friendly as possible, by using a very straightforward interface, and the option to import a scanned task that allows users to use their preferred cancellation task type. Additionally, CancellationTools supports touchscreen input, which is very comparable to pen-and-paper cancellation, for example in the sense that it allows bedside testing. Our package is open source, and is available to download for free. An online version of the task software is available to provide support for tablet devices.

## Software characteristics

### Open source

CancellationTools has been written completely in Python (Van Rossum & Drake, 2011), using as few dependencies as possible. The graphical user interface (GUI) has been written from scratch using the PyGame toolbox, and the software to analyze and visualize data has been written using the NumPy (Oliphant, 2007) and Matplotlib (Hunter, 2007) packages. All of these are open-source projects that are maintained by a large community of volunteers.

The software can be downloaded for free from www.cancellationtools.org>. It is released under the GNU General Public License version 3 (Free Software Foundation, 2007), which ensures that it can be used, shared, and modified by anyone. The source code is publicly available and managed via GitHub, which stimulates programming with frequent feedback, version control, and collaboration on a large scale – all according to the *best practices for scientific computing* as formulated by Wilson et al. (2014).

### Supported systems

A simplified version of the application can be used online. Due to copyright issues, we cannot allow users to upload their own tasks to our website. We do provide different versions of the Landolt C cancellation task. After online completion of a task, a raw data file can be downloaded, which can later be analyzed via the offline version. No data will be permanently stored or accessed by the authors of CancellationTools, or any third party. An advantage of the online runtime is that it can be accessed from computers that do not allow installation of new software (e.g., in most hospitals), or via tablet devices (e.g., Apple's iPad) that are gaining increasing popularity in neuropsychological testing.

Currently, the standalone version of CancellationTools is only available on Windows. Users of other operating systems can choose between running the application from source via Python, or using the online runtime to test participants and a PC for data analysis. We are currently working on standalone versions for other platforms, e.g., Macintosh OS X and Android, and will release these in the future.

### Interface

**Fig. 1:**
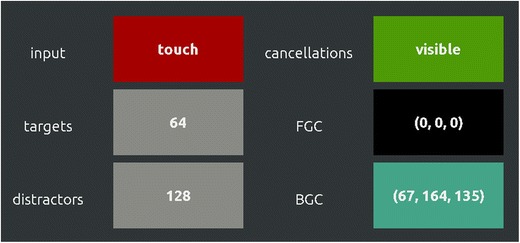
Cropped screenshot of CancellationTools' GUI, showing typical buttons and input fields from the task settings screen

We have aimed to keep the software as user-friendly as possible, without constraining functionality. The graphical user interface (GUI) is tailored to be operated smoothly via touch-screen devices and traditional PCs, and is both visually appealing and intuitive (Fig. 1). Tasks can be set up and started within a minute. Analyzing data can be done with a minimum of two mouse clicks.

### Landolt C cancellation task

**Fig. 2:**
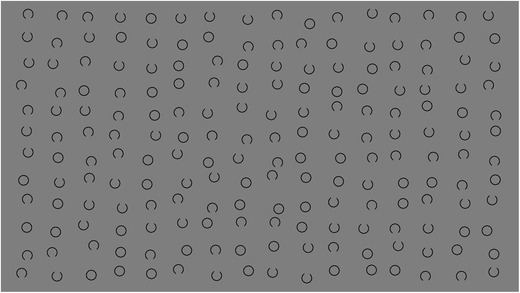
Example of a Landolt C cancellation task, where the 64 targets have a gap on top, and the 128 distractors have a gap on the bottom or no gap at all. In this example, the target to distractor ratio is 1:2. Note that targets are placed evenly over the screen width, which results in four targets per column in this example

CancellationTools’ default cancellation task is a Landolt C cancellation task, as described by Parton et al. (2006). The stimuli are circles with or without a gap, displayed in rows and columns with a random spatial jitter for each stimulus (Fig. 2). A user is free to choose the types and number of targets and distractors, the foreground and background color, the input type (mouse or touchscreen), and whether cancellation marks should be visible or not. The optimal placement of the stimuli (i.e., the number of rows and columns) is automatically calculated based on the display resolution. The placement of targets is pseudo-random, as they are placed evenly over the width of the screen. In the example task depicted in Fig. 2, this means that four targets are present in every column.

### Importing scanned tasks

For researchers and clinicians who prefer to work with a different cancellation task, CancellationTools has an option to import scanned tasks. If users select this option, they are asked to provide an image file. The image is automatically scaled to the display resolution, and a user can proceed to manually indicate where the targets and distractors are. The task is then saved, and is available for future use in task administration and analysis.

## Supported measures

We have attempted to include all of the currently existing measures that can be derived from cancellation tasks, which can be broadly divided into three categories: measures of *biases* in spatial attention, of *search organization*, and of *general performance*. Furthermore, to complement or improve on existing measures, we have devised a few of our own (e.g., the standardized angle, see below). We have not included qualitative descriptions of cancellation path structure (Samuelsson et al., 2002; Warren et al., 2008; Weintraub & Mesulam, 1988), or an algorithm to categorize search organization (Huang & Wang, 2008). In our view, these do not provide much further insight into cancellation performance than the included qualitative measures and visualizations.

### Omissions

CancellationTools reports the total number of omissions and the omissions per half of the search array, which have traditionally been used to diagnose neglect. These values are to be interpreted using standardized scores, depending on what task is employed. Traditionally, a relatively large number of omissions has been used as one index of neglect, but the left:right omissions ratio is potentially more informative and has been used widely. For example, a recent study on a particularly large sample (55 neglect patients, 138 non-neglect patients, and 119 controls) by Rabuffetti et al. (2012) reported that neglect patients show a large directional (left vs. right) imbalance in omissions, compared to healthy controls and patients with left or right lesions without neglect.

### Revisits

A revisit is a cancellation of a previously cancelled target. Some authors refer to this kind of response in the cancellation literature as '*perseveration*'. However, perseverations are often used as a term associated with a (frontal) lack of ability to inhibit. In neglect research, there is evidence that while some patients might have a problem with the ability to inhibit re-cancelling a previously visited item, others re-cancel because of a deficit in spatial working memory (Mannan et al., 2005). Therefore, we prefer to use the empirically descriptive term 'revisit'.

Revisits can occur immediately, when a participant cancels the same target twice in a row – analogous perhaps to perseveration. A delayed revisit occurs when a participant goes back to a previously cancelled target, after cancelling other targets (Mannan et al., 2005). The number of revisits correlates with measures of disorganized search, such as the best R (see below), the inter-cancellation distance, and the number of cancellation path intersections (Mark et al., 2004). Parton et al. (2006) reported that neglect patients demonstrated a higher number of revisits than non-neglect patients, an effect that was especially apparent when no cancellation marks were visible, i.e., when patients had to remember which targets they had previously visited. In this touch screen study, the median number of intervening targets was 8. The authors argued that a possible underlying mechanism for such revisiting behaviour might therefore be a deficit in spatial working memory. Our software provides the option of using an invisible cancellation condition, should users wish to use this type of search display which can provide a more sensitive measure of left:right biases in neglect, and allows investigation of the role of spatial working memory in cancellation tasks (Wojciulik et al., 2004).

### Standardized inter-cancellation distance

**Fig. 3:**
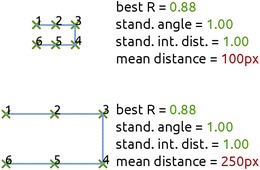
Two examples of a cancellation path. The top path was obtained from a target grid with a 100-pixel interspacing, the bottom path from a task with 250-pixel interspacing. The search organization is identical for both paths, yet the mean inter-cancellation distances are not. Other measures of search organisation (best R, standardize angle and standardized interdistance) are independent of inter-target distance

Inter-cancellation distance refers to the Euclidean distance between two consecutively cancelled targets (sometimes divided by the number of targets) and has been used to assess search behavior (Huang & Wang, 2008; Mark et al., 2004; Wang et al., 2006; Woods & Mark, 2007). We introduce a new measure that originates from the inter-cancellation distance, but is comparable across different tasks: the *standardized* inter-cancellation distance (Fig. 3). This is the mean inter-cancellation distance, divided by the mean distance between each target and its nearest neighboring target. A low standardized inter-cancellation distance originates from cancelling targets that are in close proximity of each other, and reflects an organized search pattern. Both the average and standardized inter-cancellation distance are calculated and reported by CancellationTools.

### Center of cancellation

The center of cancellation (CoC), introduced by Binder et al. (1992) and popularized by Rorden and Karnath (2010), is the average horizontal position of all cancelled targets, standardized so that a value of −1 corresponds with the leftmost, and 1 with the rightmost target. The CoC is a very elegant measure of neglect severity, as it captures an attentional gradient rather than a bimodal decision (i.e., left field is or is not impaired). Additional to the horizontal CoC., CancellationTools provides the vertical CoC, where −1 corresponds with the topmost target, and 1 with the target that is closest to the bottom of the task.

### Timing

The total amount of time a participant spends on a cancellation task might be an indication of the participant's sustained attention for the task. Primary reports indicate that this measure is potentially influenced by pharmacological intervention (Malhotra et al., 2006), and could therefore be used in diagnostics and rehabilitation. The average inter-cancellation time (sometimes dubbed *latency index*) differs between healthy controls and brain-damaged patients, but also between neglect and non-neglect patients (Rabuffetti et al., 2012). It could hypothetically serve as a measure of executive functioning, as it reflects how much processing time a participant needs to find and cancel a new target.

### Search speed

The search speed is the average of all inter-cancellation distances divided by all inter-cancellation times (Eq. 1), and has been introduced and validated by Rabuffetti et al. (2012), who show that controls are slightly faster than brain-damaged patients. This is not surprising, as the same study reports lower inter-cancellation times for patients than for controls.

1 $$ {v}_{search}=\frac{{\displaystyle \sum_{i=1}^{n-1}\frac{s_i}{t_i}}}{n-1} $$

Where:

*n* is the number of cancellations

*s* is the distance between two consecutive cancellations

*t* is the time between two consecutive cancellations

### Quality of search (Q) score

A measure of the quality of search, is the Q score introduced by Hills and Geldmacher (1998). The Q score combines speed and accuracy in a single measure, and is calculated using Eq. 2. A high Q score reflects a combination of a high number of cancelled targets, and a high cancellation speed. This index does not seem to be task independent: Huang & Wang (2008) found that Q scores in healthy undergraduates were higher for unstructured arrays compared to structured arrays. The number of correct responses for both task types did not differ, meaning that the difference in Q scores was driven by a higher time-on-task for the structured array. However, one should be careful when interpreting these results, as the terms 'structured' and 'unstructured' only applied to the distractors in this study: The targets locations were the same for both tasks, and only the distractors (the noise) were distributed either with or without equal spacing.

2 $$ Q=\frac{{N_{cor}}^2}{N_{tar}\cdot {t}_{tot}} $$

Where:

*N*_*cor*_ is the number of cancelled targets (correct responses)

*N*_*tar*_ is the total number of targets

*t*_*tot*_ is the total time spent on the task

### Intersections rate

**Fig. 4:**
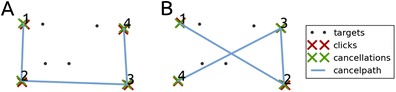
Examples of cancellation paths. (**a**) A path that does not cross itself, resulting in no intersections and an intersection rate of 0. (**b**) A path that does cross itself, resulting in 1 intersection and an intersection rate of 0.25

Donnelly et al. (1999) counted the total number of cancellation path intersections: the number of times a cancellation path crosses itself (Fig. 4). Mark et al. (2004) and Rabuffetti et al. (2012) divided the intersections total by the amount of produced markings to correct for search path length, resulting in the *intersections rate*. Rabuffetti et al. use the term *crossing index*, which differs from the intersections rate in one aspect: The total amount of intersections is divided by the total amount of markings, whereas the intersections rate of Mark et al. is calculated by dividing the amount of intersections by the total amount of markings excluding immediate revisits. As the cancellation path is only determined by cancelled targets, we define the intersections rate as the total amount of path intersections divided by the amount of cancellations that are not immediate revisits (Eqs. 3–8).

An efficient search pattern includes as few intersections as possible. In other words, a high rate of intersections would be indicative for unsystematic exploration. Rabuffetti et al. (2012) have shown that this measure can differentiate between different groups of participants: controls < non-neglect right-brain damage < non-neglect left-brain damage < neglect right-brain damage.

3 $$ {D}_x=\left({x}_{1,i}\cdot {y}_{2,i}-{y}_{1,i}\cdot {x}_{2,i}\right)\cdot \left({x}_{1,j}-{x}_{2,j}\right)-\left({x}_{1,i}-{x}_{2,i}\right)\cdot \left({x}_{1,j}\cdot {y}_{2,j}-{y}_{1,j}\cdot {x}_{2,j}\right) $$

4 $$ {D}_y=\left({x}_{1,i}\cdot {y}_{2,i}-{y}_{1,i}\cdot {x}_{2,i}\right)\cdot \left({y}_{1,j}-{y}_{2,j}\right)-\left({y}_{1,i}-{y}_{2,i}\right)\cdot \left({x}_{1,j}\cdot {y}_{2,j}-{y}_{1,j}\cdot {x}_{2,j}\right) $$

5 $$ D=\left({x}_{1,i}-{x}_{2,i}\right)\cdot \left({y}_{1,j}-{y}_{2,j}\right)-\left({y}_{1,i}-{y}_{2,i}\right)\cdot \left({x}_{1,j}-{x}_{2,j}\right) $$

6 $$ \left({P}_x,{P}_y\right)=\left(\frac{D_x}{D},\frac{D_y}{D}\right) $$

7 $$ {N}_{intersect}={\displaystyle \sum_{i=1}^{n-1}{\displaystyle \sum_{j=i+1}^{n-1}\left(D>0\right)\wedge \left(\left({x}_{1,j}<{P}_x<{x}_{2,i}\right)\wedge \left({x}_{1,j}<{P}_x<{x}_{2,j}\right)\right)\wedge \left(\left({y}_{1,i}<{P}_y<{y}_{2,i}\right)\wedge \left({y}_{1,j}<{P}_y<{y}_{2,j}\right)\right)}} $$

8 $$ {r}_{intersect}=\frac{N_{intersect}}{N_{cancellation}-{N}_{imm. revisit}} $$

Where:

(*X*_*1*_ , *y*_*1*_) is the starting coordinate of the line between two consecutive cancellations (cancellation *n*)

(*X*_*2*_ , *y*_*2*_) is the ending coordinate of the line between two consecutive cancellations (cancellation *n*+*1*)

(*P*_*x*_ , *P*_*y*_) is the coordinate of the intersection between two inter-cancellation lines

*n* is the number of inter-cancellation lines (not to be confused with the number of cancellations)

### Best R

**Fig. 5:**
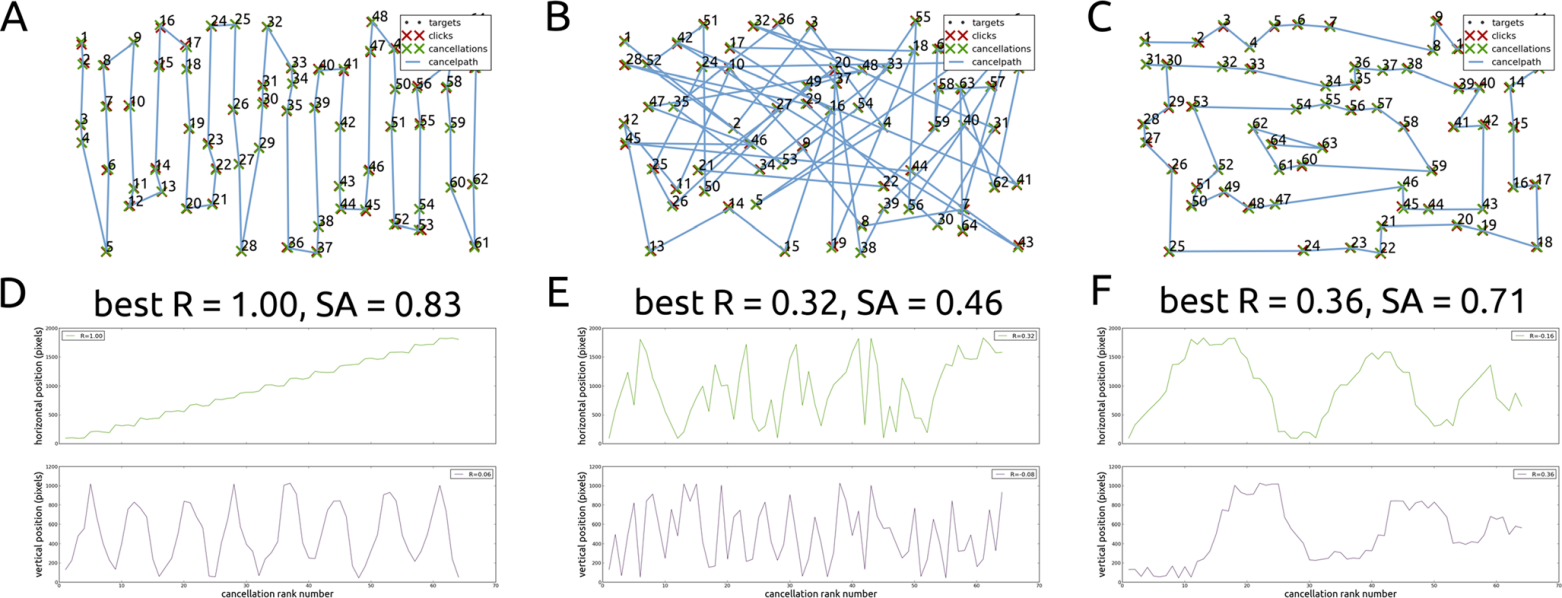
Three search paths to illustrate the best R and the standardized angle. (**a**) An efficient search, starting on the left of the field and proceeding in a general rightward direction, results in a high correlation between the cancellation rank number and the horizontal cancellation position. (**b**) An inefficient search has no general direction, and leads to low correlations between the cancellation rank number and both the horizontal and vertical cancellation position. (**c**) Another efficient search path, resulting in a lower best R, but a high standardized angle. (**d**-**f**) Best R plots of cancellation paths **a**-**c**

Mark et al. (2004) coined a quantitative measure for assessing cancellation strategy, which can be viewed as a formalization of the qualitative ways in which some researchers have tried to describe cancellation paths (Samuelsson et al., 2002; Warren et al., 2008; Weintraub & Mesulam, 1988). The best R is defined as the highest absolute value of the Pearson correlation between cancellation rank number and either horizontal or vertical cancellation position (Eq. 9, Fig. 5), and should increase with search efficiency. The most efficient way of performing a cancellation task is to start searching at an extremity (e.g., the left), and proceed with the search in one general direction (e.g., rightward or downward), alternating moving up and down on the perpendicular direction (e.g., upward and downward, or leftward and rightward), as is depicted in Fig. 5a.

9 $$ {R}_{best}= \max \left(\left|{R}_{hor}\right|,\left|{R}_{ver}\right|\right) $$

Where:

*R*_*hor*_ is the Pearson correlation coefficient of the horizontal position of all cancellations and their rank numbers

*R*_*ver*_ is the Pearson correlation coefficient of the vertical position of all cancellations and their rank numbers

### Standardized angle

**Fig. 6:**
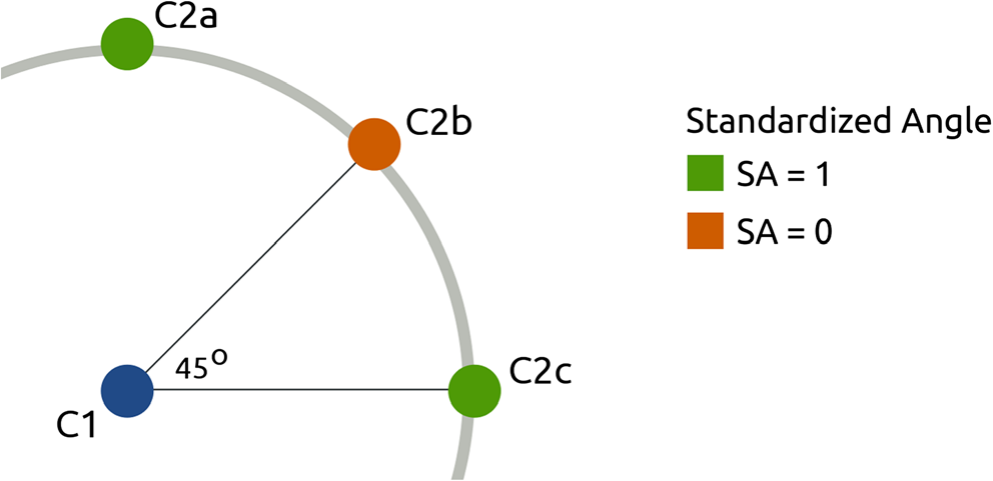
Illustration of the standardized angle. C1 is a cancelled target, C2a-c are potential consecutive cancellations. The standardized angle is 1 for vertical (between C1 and C2a) or horizontal (between C1 and C2c) inter-cancellation angles, and approaches 0 for diagonal angles (between C1 and C2b). Paths containing predominantly horizontal and vertical lines are considered to be more efficient, therefore a high standardized angle is an indication of an efficient search pattern

One of the possible cancellation paths that is efficient, but will nonetheless result in a relatively low best R, is a circular path that starts in the extremes of the cancellation task, and gradually moves inward, or spirals (Fig. 5c). What characterizes this kind of path and the paths that do result in a high best R (e.g., Fig. 5a) is the occurrence of predominantly horizontal and vertical lines between cancellation locations. We introduce a measure that can differentiate between horizontal and vertical paths (associated with an optimal search strategy) on the one hand, and diagonal lines (associated with a suboptimal strategy) on the other (Eqs. 10 and 11). As the inter-cancellation angle approaches 45°, the standardized angle approaches 0. In contrast, inter-cancellation angles approaching either 90° or 0° will result in a standardized angle that approaches 1 (Fig. 6). Therefore, a high standardized angle is potentially an indication of an efficient cancellation process.

10 $$ \gamma = \arcsin \left(\frac{\varDelta_y}{d}\right) $$

11 $$ {\gamma}_{standardized}=\frac{{\displaystyle \sum_{i=1}^n|\frac{2\cdot {\gamma}_i}{90}-1}|}{n} $$

Where:

*γ* is the angle between two consecutive cancellations

*Δy* is the vertical distance between two consecutive cancellations

*d* is the Euclidean distance between two consecutive cancellations

*n* is the total amount of inter-cancellation angles between consecutive cancellations that are not immediate revisits

### First marking

Age has a significant influence on measures of search organisation. Specifically, the mean inter-cancellation distance and the amount of intersections decrease as age increases in children, while the best R increases, demonstrating an improvement in search organisation over time (Woods et al., 2013). Another index that increases with age is the likelihood of the first cancellation to be in the top-left quadrant of the search array. CancellationTools provides the location of the first marking in standardized space, so that the top left of the search array is (0,0) and the bottom right (1,1). These standardized locations are comparable between different task types and sizes. A qualitative description (e.g., “top-left”) of the quadrant in which the first cancellation happened is also available.

### Overview

To give a preliminary indication of the ranges of the summarized cancellation measures, we tested small samples of healthy adults (*N* = 10) and right-hemisphere patients with leftward neglect (*N* = 10). They were tested on Landolt C cancellation tasks that consisted of 64 targets (opening on top) and 128 distractors (50 % without opening, and 50 % with an opening on the bottom), on which cancellation markings were invisible, and the time limit was 2 min. The averages, standard deviations, and 95 % confidence intervals of all CancellationTools' quantitative measures are listed in Table 1. These values should not be regarded as norm scores. More elaborate studies on larger samples include Rabuffetti et al. (2012) (omissions, revisits, inter-cancellation distance and time, cancellation speed, and amount of path intersections in healthy controls, stroke patients with and without neglect), Woods & Mark (2007) (inter-cancellation distance, intersection rate, and best R in a healthy and a non-neglect stroke patient sample), Parton et al. (2006) (immediate and delayed revisits in stroke patients with and without neglect), and Rorden & Karnath (2010) (center of cancellation in neglect and non-neglect patients with right hemisphere damage).

**Table 1:** Averages, standard deviations (between round brackets), and 95 % confidence intervals (between square brackets) of a healthy sample and a neglect patient sample, collected using 1280×1024 pixels Landolt C cancellation tasks with 64 targets and 128 distractors, invisible cancellation markings, and a time limit of 2 min

	Healthy sample (*N*=10)	Neglect sample (*N*=10)
Omissions (total)	2.5 (2.0)	41.6 (12.6)
	[1.3, 3.7]	[33.8, 49.4]
Omissions in left half	1.4 (1.6)	27 (6.6)
	[0.4, 2.4]	[22.9, 31.1]
Omissions in right half	1.1 (1.2)	14.6 (7.3)
	[0.4, 1.8]	[10.1, 19.1]]
Revisits (total)	2.3 (3.7)	27.8 (18.3)
	[0.0, 4.6]	[16.5, 39.1]
Immediate revisits	0.6 (1.1)	12 (20.7)
	[-0.1, 1.3]	[-0.8, 24.8]
Delayed revisits	1.7 (2.7)	15.8 (13.3)
	[0.0, 3.4]	[7.5, 24.1]
Horizontal center of cancellation	0.04 (0.02)	0.55 (0.31)
	[0.03, 0.06]	[0.36, 0.75]
Vertical center of cancellation	0.13 (0.03)	0.01 (0.11)
	[0.11, 0.14]	[-0.06, 0.07]
Task duration (sec.)	76.2 (14.3)	116.1 (6.9)
	[67.3, 85.1]	[111.8, 120.4]
Mean inter-cancellation time (sec.)	1.17 (0.19)	2.48 (0.87)
	[1.06, 1.29]	[1.95, 3.02]
Q score	0.80 (0.14)	0.09 (0.08)
	[0.71, 0.89]	[0.04, 0.14]
Mean inter-cancellation distance (pixels)	210.6 (32.7)	235.0 (40.6)
	[190.3, 230.9]	[209.9, 260.2]
Standardized inter-cancellation distance	2.30 (0.40)	2.57 (0.48)
	[2.05, 2.55]	[2.28, 2.87]
Speed (cancellations per second)	0.18 (0.03)	0.11 (0.04)
	[0.16, 0.20]	[0.08, 0.13]
Mean inter-cancellation angle	41.1 (29.8)	57.1 (13.0)
	[22.6, 59.5]	[49.0, 65.2]
Standardized inter-cancellation angle	0.74 (0.07)	0.64 (0.07)
	[0.70, 0.78]	[0.59, 0.68]
Best R	0.99 (0.01)	0.50 (0.29)
	[0.99, 0.99]	[0.32, 0.68]
Path intersections	1.2 (2.1)	19.3 (20.5)
	[-0.1, 2.5]	[6.6, 32.0]
Intersection rate	0.02 (0.04)	0.44 (0.43)
	[0.00, 0.04]	[0.18, 0.71]
First cancellation x-coordinate (standardized space)	0.03 (0.00)	0.77 (0.20)
	[0.03, 0.03]	[0.64, 0.89]
First cancellation y-coordinate (standardized space)	0.06 (0.01)	0.32 (0.17)
	[0.06, 0.06]	[0.22, 0.43]

Theoretically task-independent measures (provided there is a relatively equal spread of targets over the search array) are left:right omission ratio, standardized inter-cancellation distance, center of cancellation, average inter-cancellation speed, intersections rate, and location of the first cancellation in standardized space. Whether this theoretical task-independency holds up in practice, might be determined in future research.

## Output

### Summarized measures

**Fig. 7:**
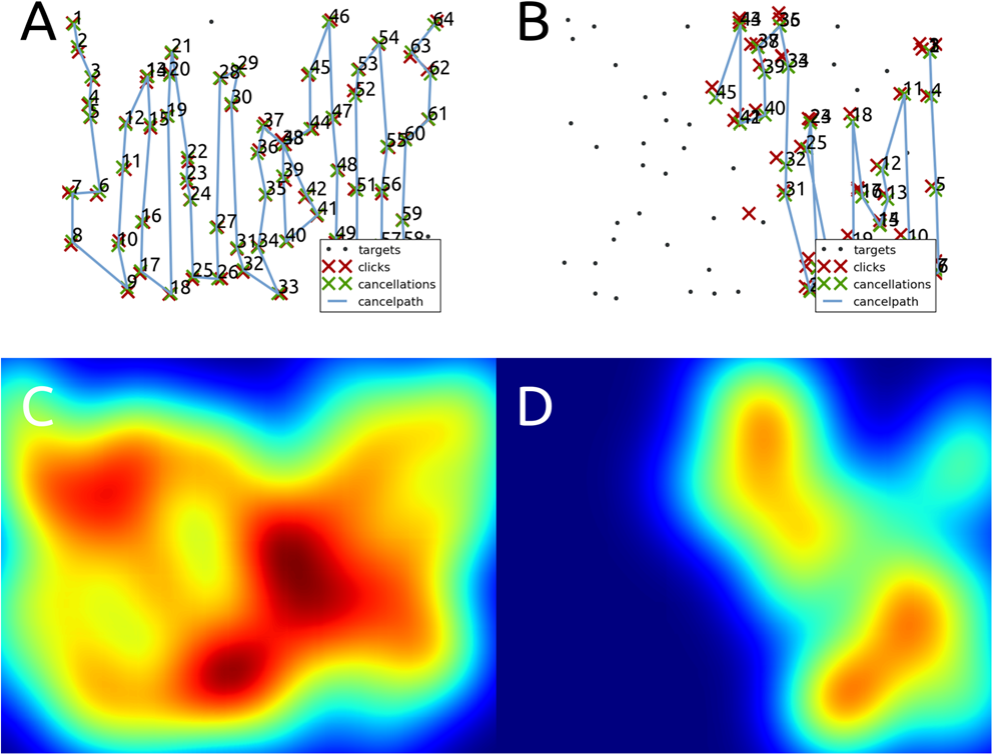
Output files of two example data sets, collected on a Landolt C cancellation task with invisible cancellation markings. (**a**) A cancellation path of a typical healthy adult. (**b**) The cancellation path of a patient suffering from severe leftward neglect. (**c** and **d**) Heatmaps of the cancellation path presented in **a** and **b**

Two kinds of summarized results are produced. The first is a single A4-sized, high-quality PDF document that contains an overview of all outcome measures, as well as a plot of the cancellation path (Fig. 7a-b) and a heatmap of the cancelled targets (Fig. 7c-d). This kind of output is potentially useful in a clinical setting, where a medical professional can consecutively run a task and an analysis, and add a print of the results to a patient’s file. Furthermore, a simple text file is created, which can be opened with spreadsheet software (e.g., OpenOffice Calc or Microsoft Excel) and statistics packages (e.g., PSPP or SPSS), and can easily be processed using custom analysis scripts (e.g., using Python, R, Matlab, or any other programming language). Using the text files, researchers can easily extract data from a large group of participants for further analysis. Also available is an image of the cancellation task with all cancellation markings that a participant made (i.e., as the participant saw the task upon finishing), and a text file of all raw click (or touch) times and coordinates.

### Data visualization

Several plots are created by each CancellationTools analysis. These give further insight into the performance of single participants, and can be used in addition to the measures described above. These plots include the aforementioned cancellation path and heatmap. The cancellation path (Fig. 7a-b) gives a clear view of a participant's cancellation behavior, e.g., to help with the interpretation of measures of disorganized search. A plot of the relation between the cancellation rank number and either the horizontal or vertical position of the cancelled target (Fig. 5d-f) gives an indication of how organized a participants search was (Mark et al., 2004; Woods & Mark, 2007).

**Fig. 8:**
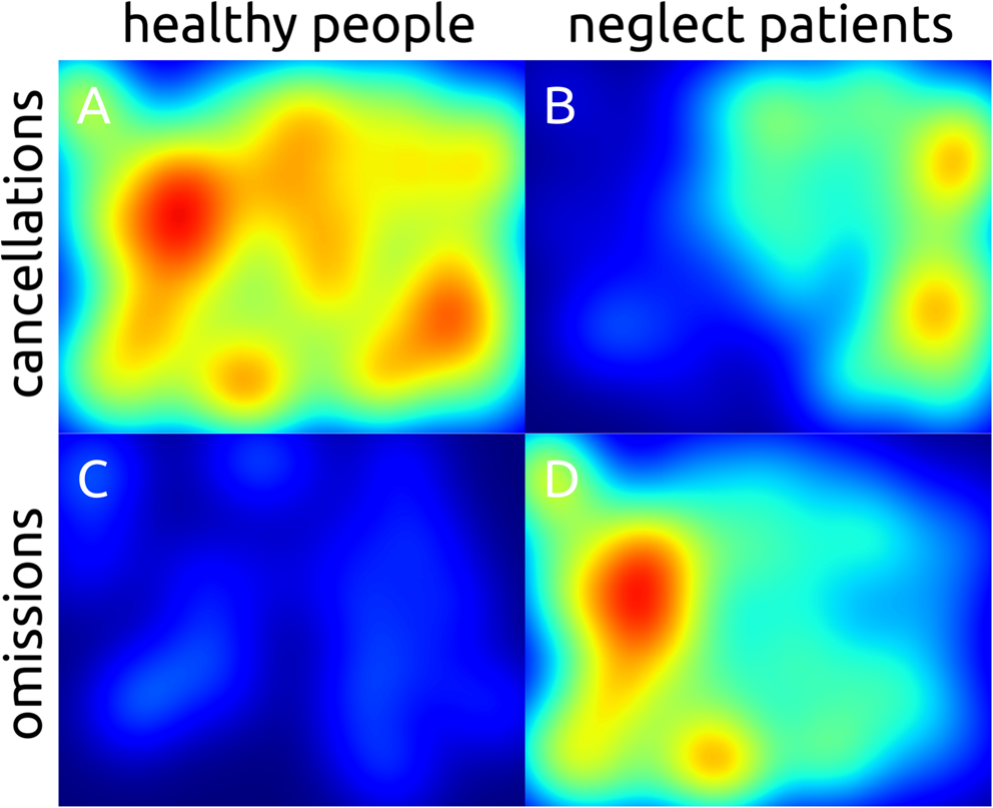
Cancellation heatmaps (**a**-**b**) and omission heatmaps (**c**-**d**) for a healthy sample (N=10, **a** and **c**), and a sample of right-hemisphere, leftward neglect patients (N=10, **b** and **d**). The data was collected using 1280×1024 pixels Landolt C cancellation tasks with 64 targets and 128 distractors, invisible cancellation markings, and a time limit of 2 min

Heatmaps of fixation locations illustrate the deployment of attention in 2D-space, as is demonstrated by Bays et al. (2010). With our cancellation heatmaps we aim to create a similar visualization of spatial attention. Our pilot testing is promising on both an individual level (Fig. 7c-d), and on a group level (Fig. 8). Additional heatmaps are provided based on the locations of omissions, and on the locations of path intersections, to give an indication of the spatial properties of these measures.

For the cancellation and omission a Gaussian kernel is added to the location of each cancelled or missed target. The resulting field is then scaled to the heatmap that would result from an optimal performance on the cancellation task in question, which means that heatmaps are comparable between individuals and tasks. Heatmaps for individual data from a healthy individual and a neglect patient are displayed in Fig. 7c-d. Averaged heatmaps of a healthy and a neglect sample are shown in Fig. 8, and show an even spread of cancellations across the search array in healthy people (Fig. 8a), whereas neglect patients show a rightward bias (Fig. 8b). Neglect patients also display a leftward bias of omissions (Fig. 8d), whereas our healthy sample shows a lack of omissions (Fig. 8c).

## Discussion

There is a need to quantify multitarget visual search performance on cancellation tasks. We made an effort to summarize all of the currently available measures that can be derived from cancellation task data. In the new software introduced here, we included all relevant measures from the currently available literature in an application that can be used to administer a computerized cancellation task, and to analyze the resulting data with the click of a button. We have aimed to make this software as flexible as possible, e.g., by allowing users to incorporate their own scanned tasks into the software, whilst keeping an eye on simplicity. The result is a user-friendly interface that can be employed both in clinical and research settings. Our software is open source, and free to download and use by anyone.

We have introduced two new measures of search organisation: the standardized inter-cancellation distance and angle. The former is an improvement of the existing mean inter-cancellation distance, which takes into account the distances between targets within a search array, therefore allowing comparisons of cancellation performance on different tasks. The standardized inter-cancellation angle can be viewed as complimentary to the best R, as it is robust to situations where the best R does not reflect search organisation optimally (Fig. 5c). Even though the best R and standardized inter-cancellation angle seem to differentiate between our small test groups, a much larger difference between healthy people and leftward neglect patients is observed in the intersections rate, suggesting that this might be the clearest measure of search organisation.

CancellationTools is already useful to clinicians, as it provides quantitative data on established measures of neglect (e.g., number of omissions), as well as qualitative data that provides better insight in patient behavior than pen-and-paper cancellation tests (e.g., cancellation path plots). However, for the majority of the measures summarized above, there are currently no norm scores to compare individual test results to. The value ranges that we provide based on our pilot testing (Table 1) serve as a preliminary indication of how neglect patients and healthy controls differ on different measures, and should not be treated as a clinical directive.

Apart from our newly introduced standardized angle measure, all of the indices we report have been validated on a small scale in the articles in which they were coined. A few have been validated on a larger scale in the study of Rabuffetti et al. (2012), but it is arguable whether this provides enough data to base norm scores on. We aim to facilitate the fast testing of the summarized measures by providing a unified tool that helps to gather cancellation task data as easy as possible. Our hope is that this will help to establish norm scores for the measures that proof to have diagnostic value.

By making CancellationTools publicly available, we hope to inspire large-scale international collaborations to pool data, from healthy people and patient groups, on all of the measures we summarize in the current article. By removing practical boundaries that previously prevented large-scale testing, our software opens up exciting new research possibilities. The availability of CancellationTools creates a situation in which analysis of cancellation task data can be performed at a high level across different clinical and research settings.

## References

[CR1] Appelros P, Karlsson GM, Seiger A, Nydevik I (2002). Neglect and Anosognosia After First-Ever Stroke: Incidence and Relationship to Disability. Journal of Rehabilitation Medicine.

[CR2] Bays PM, Singh-Curry V, Gorgoraptis N, Driver J, Husain M (2010). Integration of Goal- and Stimulus-Related Visual Signals Revealed by Damage to Human Parietal Cortex. Journal of Neuroscience.

[CR3] Binder J, Marshall R, Lazar R, Benjamin J, Mohr JP (1992). Distinct Syndromes of Hemineglect. Archives of Neurology.

[CR4] Buxbaum, L. J., Ferraro, M. K., Veramonti, T., Farne, A., Whyte, J., Ladavas, E., … Coslett, H. B. (2004). Hemispatial neglect: Subtypes, neuroanatomy, and disability. *Neurology*, *62*(5), 749–756. doi:10.1212/01.WNL.0000113730.73031.F4 10.1212/01.wnl.0000113730.73031.f4 15007125

[CR5] Donnelly N, Guest R, Fairhurst M, Potter J, Deighton A, Patel M (1999). Developing algorithms to enhance the sensitivity of cancellation tests of visuospatial neglect. Behavior Research Methods, Instruments, & Computers.

[CR6] Fabrigoule, C., Rouch, I., Taberly, A., Letenneur, L., Commenges, D., Mazaux, J. M., … Dartigues, J. F. (1998). Cognitive process in preclinical phase of dementia. *Brain*, *121*(1), 135–141. doi:10.1093/brain/121.1.135 10.1093/brain/121.1.135 9549494

[CR7] Free Software Foundation. (2007). GNU General Public License. Retrieved from https://gnu.org/licenses/gpl.html

[CR8] Gainotti G, Marra C, Villa G (2001). A double dissociation between accuracy and time of execution on attentional tasks in Alzheimer’s disease and multi-infarct dementia. Brain.

[CR9] Hills EC, Geldmacher DS (1998). The effect of character and array type on visual spatial search quality following traumatic brain injury. Brain Injury.

[CR10] Huang H-C, Wang T-Y (2008). Visualized representation of visual search patterns for a visuospatial attention test. Behavior Research Methods.

[CR11] Hunter JD (2007). Matplotlib: A 2D Graphics Environment. Computing in Science & Engineering.

[CR12] Husain M, Rorden C (2003). Non-spatially lateralized mechanisms in hemispatial neglect. Nature Reviews. Neuroscience.

[CR13] Lesniak M, Bak T, Czepiel W, Seniow J, Czlonkowska A (2008). Frequency and Prognostic Value of Cognitive Disorders in Stroke Patients. Dementia and Geriatric Cognitive Disorders.

[CR14] Malhotra PA, Parton AD, Greenwood R, Husain M (2006). Noradrenergic modulation of space exploration in visual neglect. Annals of Neurology.

[CR15] Mannan SK, Mort DJ, Hodgson TL, Driver J, Kennard C, Husain M (2005). Revisiting Previously Searched Locations in Visual Neglect: Role of Right Parietal and Frontal Lesions in Misjudging Old Locations as New. Journal of Cognitive Neuroscience.

[CR16] Mark VW, Woods AJ, Ball KK, Roth DL, Mennenmeier M (2004). Disorganized search on cancellation is not a consequence of neglect. Neurology.

[CR17] Nijboer TCW, Kollen BJ, Kwakkel G (2013). Time course of visuospatial neglect early after stroke: A longitudinal cohort study. Cortex.

[CR18] Nijboer TCW, Kollen BJ, Kwakkel G (2014). The Impact of Recovery of Visuo-Spatial Neglect on Motor Recovery of the Upper Paretic Limb after Stroke. PLoS ONE.

[CR19] Nijboer, T. C. W., Van de Port, I., Schepers, V., Post, M., & Visser-Meily, A. (2013b). Predicting Functional Outcome after Stroke: The Influence of Neglect on Basic Activities in Daily Living. *Frontiers in Human Neuroscience*, *7*. doi:10.3389/fnhum.2013.00182 10.3389/fnhum.2013.00182 PMC3650314 23675336

[CR20] Nys GMS, Van Zandvoort MJE, De Kort PLM, Van der Worp HB, Jansen BP, De Haan EHF, Kappelle LJ (2005). The prognostic value of domain-specific cognitive abilities in acute first-ever stroke. Neurology.

[CR21] Oliphant TE (2007). Python for Scientific Computing. Computing in Science & Engineering.

[CR22] Parton AD, Malhotra PA, Nachev P, Ames D, Ball J, Chataway J, Husain M (2006). Space re-exploration in hemispatial neglect. NeuroReport.

[CR23] Rabuffetti, M., Farina, E., Alberoni, M., Pellegatta, D., Appollonio, I., Affanni, P., … Ferrarin, M. (2012). Spatio-Temporal Features of Visual Exploration in Unilaterally Brain-Damaged Subjects with or without Neglect: Results from a Touchscreen Test. *PLoS ONE*, *7*(2), e31511. doi:10.1371/journal.pone.0031511 10.1371/journal.pone.0031511 PMC3275551 22347489

[CR24] Ringman JM, Saver JL, Woolson RF, Clarke WR, Adams HP (2004). Frequency, risk factors, anatomy, and course of unilateral neglect in an acute stroke cohort. Neurology.

[CR25] Rorden C, Karnath H-O (2010). A simple measure of neglect severity. Neuropsychologia.

[CR26] Samuelsson H, Hjelmquist EKE, Jensen C, Blomstrand C (2002). Search pattern in a verbally reported visual scanning test in patients showing spatial neglect. Journal of the International Neuropsychological Society.

[CR27] Van Rossum G, Drake FL (2011). Python Language reference manual.

[CR28] Wang T-Y, Huang H-C, Huang H-S (2006). Design and implementation of cancellation tasks for visual search strategies and visual attention in school children. Computers & Education.

[CR29] Warren M, Moore JM, Vogtle LK (2008). Search Performance of Healthy Adults on Cancellation Tests. American Journal of Occupational Therapy.

[CR30] Weintraub S, Mesulam M-M (1988). Visual hemispatial inattention: stimulus parameters and exploratory strategies. Journal of Neurology, Neurosurgery & Psychiatry.

[CR31] Wilson, G., Aruliah, D. A., Brown, C. T., Chue Hong, N. P., Davis, M., Guy, R. T., … Wilson, P. (2014). Best Practices for Scientific Computing. *PLoS Biology*, *12*(1), e1001745. doi:10.1371/journal.pbio.1001745 10.1371/journal.pbio.1001745 PMC3886731 24415924

[CR32] Wojciulik E, Rorden C, Clarke K, Husain M, Driver J (2004). Group study of an “undercover” test for visuospatial neglect: invisible cancellation can reveal more neglect than standard cancellation. Journal of Neurology, Neurosurgery & Psychiatry.

[CR33] Woods AJ, Göksun T, Chatterjee A, Zelonis S, Mehta A, Smith SE (2013). The development of organized visual search. Acta Psychologica.

[CR34] Woods AJ, Mark VW (2007). Convergent validity of executive organization measures on cancellation. Journal of Clinical and Experimental Neuropsychology.

